# Music Regulators in Two String Quartets: A Comparison of Communicative Behaviors between Low- and High-Stress Performance Conditions

**DOI:** 10.3389/fpsyg.2016.01229

**Published:** 2016-08-25

**Authors:** Michele Biasutti, Eleonora Concina, David Wasley, Aaron Williamon

**Affiliations:** ^1^Dipartimento di Filosofia, Sociologia, Pedagogia e Psicologia Applicata, Padova UniversityPadova, Italy; ^2^Cardiff School of Sport, Cardiff Metropolitan UniversityCardiff, UK; ^3^Centre for Performance Science, Royal College of MusicLondon, UK

**Keywords:** music regulators, behavioral coordination, eye contact, video analysis, string quartet ensembles

## Abstract

In ensemble performances, group members use particular bodily behaviors as a sort of “language” to supplement the lack of verbal communication. This article focuses on music regulators, which are defined as signs to other group members for coordinating performance. The following two music regulators are considered: body gestures for articulating attacks (a set of movements externally directed that are used to signal entrances in performance) and eye contact. These regulators are recurring observable behaviors that play an important role in non-verbal communication among ensemble members. To understand how they are used by chamber musicians, video recordings of two string quartet performances (*Quartet A* performing Bartók and *Quartet B* performing Haydn) were analyzed under two conditions: a low stress performance (LSP), undertaken in a rehearsal setting, and a high stress performance (HSP) during a public recital. The results provide evidence for more emphasis in gestures for articulating attacks (i.e., the perceived strength of a performed attack-type body gesture) during HSP than LSP. Conversely, no significant differences were found for the frequency of eye contact between HSP and LSP. Moreover, there was variability in eye contact during HSP and LSP, showing that these behaviors are less standardized and may change according to idiosyncratic performance conditions. Educational implications are discussed for improving interpersonal communication skills during ensemble performance.

## Introduction

Chamber musicians use high levels of task and group awareness to synchronize their performance. This process is founded on implicit rules and is a sort of language composed of behaviors that overcome the inability to communicate verbally. It is difficult to say when or if these behaviors are standardized or used idiosyncratically by performers and how these vary across different performance situations, such as rehearsals and recitals. In order to explore these points, the present study analyzed how two string quartets used these behaviors, termed music regulators, between group members and across performance situations. Music regulators are gestures that achieve communication during ensemble performance (Davidson, 2001; Davidson and Correia, 2002; Davidson and King, 2004). They are mainly used with the aim of synchronizing individual performances, particularly emphasizing music entrances of group members, time beating, feedback, and the ends of musical phrases. They are intended to support the coordination of individual performances into a global and holistic musical result.

### Music Gestures and Communication: Regulators in Music Performance

There is a growing interest in research that considers gesture and behavioral coordination among musicians during music performance (Davidson and Correia, 2002; Williamon and Davidson, 2002; Davidson and King, 2004; Kurosawa and Davidson, 2005). The purposes of such movements and gestures appear to have three specific functions: (1) to ensure the correct sound production, (2) to contribute to musical expression, and (3) to support interpersonal communication within a social context (Davidson and Correia, 2002; Davidson and Good, 2002; Davidson and King, 2004). The first function is internally orientated and depends on the particular technique of the musical instrument played. The other two refer to gestures that are externally directed. The social context of music performance requires performers to consider the audience experience. This can take place in any public performance, where the resultant musical outcomes are supported and amplified by appropriate gestural expressiveness. Therefore, musicians’ bodily gestures can take on different meanings: several studies have analyzed the external communicative role that performers’ bodily gestures take on, focusing on soloists (Davidson, 2001, 2012), duos (Davidson, 2012), and other ensemble performance (Davidson and Good, 2002; Kurosawa and Davidson, 2005). There are three main kinds of externally oriented gestures (Davidson, 2001, 2005): “illustrators” (self-explanatory gestures of emphasis); “emblems” (gestural symbols, with cultural and social meaning); and “regulators” (gestures used to mark entrances and exits) which were developed on the non-verbal communication research of Ekman and Friesen (1969). For the purpose of this study, regulators are the principle communications of interest. Therefore different types of communicative behaviors are recognized according to their function:

“[Regulators] *are acts which maintain and regulate the back-and-forth nature of speaking and listening between two or more interactants (*…) *The regulators (*…*) are related to the conversational flow, the pacing of the exchange.”* (Ekman and Friesen, 1969, p. 82).

This definition highlights the role of non-verbal behaviors in enhancing verbal communication. Since verbal exchanges are not possible during musical performance, regulators may assume a greater role in co-performers’ activities; they have to be clear and precise bodily gestures, such as nodding with the head, beating the time with the hand or the feet, and/or exchanging eye contact. Moreover, they have to be distinguishable from gestures connected to internal functions (e.g., sound production and expressive gestures) and understood by the other performers as such.

### Research about Regulators in Chamber Ensembles and String Quartets

Unlike soloists, ensemble performers have to develop a dual communicative process: in addition to the communication of musical meaning with the audience, they have to develop a process of musical synchronization with co-performers (Davidson, 2005; Lehmann et al., 2007), intended to overcome the inability to communicate verbally (Davidson, 1997). In ensembles, visual elements have a relevant role in synchronizing the whole performance (McPherson and Schubert, 2004), and supporting the information conveyed by auditory feedback. Research examining communication specifically in chamber ensembles has used piano duos (Williamon and Davidson, 2002). The results of this were that, as the pianists became more familiar with the piece over the practice sessions, there was an increase in the frequency of gestures and eye contact externally orientated that was maintained during the final performance. In addition, Davidson (2012) considered the role of body gesture in musical expressiveness in different performance conditions (solo and ensemble performance) for wind players. With a qualitative analysis, specific categories of gestures were identified, each of them conveying a specific expressive meaning. In duo ensembles, gestures are used with the aim of coordinating individual performances; eye contact has a function of supporting coordination but was less frequent than expected based on other studies. With regard to string quartet ensembles, studies have considered different issues, such as leadership, individual roles, and the social dimension, while only a few have focused on musical communication (Davidson and Good, 2002; Seddon and Biasutti, 2009a,b).

In a case study with a student string quartet, Davidson and Good (2002) used video recordings and interviews to illustrate how several factors influence musical coordination, including social-cultural, socio-emotional, and personal characteristics, as well as performance anxiety and distractors. There was evidence during the rehearsals that the first violin was observed by all the other members, while the first violinist directed more attention to the cellist and second violinist. Considering the directions of eye contact, some main communicative patterns were identified, such as the visual exchange between the first violinist and the cellist, and between the second violinist and the violist. In addition, the students’ restricted experience in performing in a string quartet was considered a factor that limited their communication to individual and technical issues rather than to more creative content. Seddon and Biasutti (2009a,b) considered the modes of communication employed between members of a professional string quartet identifying the following six modes of communication: instruction, cooperation, and collaboration both for verbal and non-verbal behaviors. Body language and elements, such as eye contact and musical cues, were considered essential aspects allowing musicians to produce a cohesive performance.

### Different Performance Conditions: Rehearsal versus Concert Performance

The performance condition is an important variable since, during both rehearsals and concerts, musicians have to control many contextual and individual aspects. While the rehearsal is an event characterized by a variable level of stress, in the concert musicians must always face the stress of playing in front of an audience (Davidson and Correia, 2001; Williamon et al., 2013). This research considered the differences between rehearsing and performing in concert and level of stress during different performance conditions.

With regard to the differences between rehearsing and performing in concert, Davidson and Correia (2001) analyzed the building of communicative processes. With a qualitative inquiry, they considered rehearsal strategies and the role of body movement and gesture in shaping musical communication between the performer and the audience. According to the authors, there are fundamental differences between rehearsal and performance, namely that the presence of an audience emphasizes the communicative dimension of musical performance and thereby affects performers’ gestures. Gestures express musical meanings through performers’ bodily experiences of the world. Gestures directly affect audiences’ perceptions of music: they might be considered more as “metaphorical projections” (p. 80) than a formal codified communicative system.

Regarding the stress during different performance conditions LeBlanc et al. (1997) found a significant increase of self-report perceived anxiety of students performing solos under three levels of audience presence: performing alone in a practice room, in a practice room with one researcher, and in the rehearsal room with more people. Self-reported anxiety rose with each successive performance condition, and each reported increase was significant. Williamon et al. (2013) used performing in front of an audience as a high stress condition and analyzed the physical consequences that derived from it. They assessed the heart rate variability (HRV) of an expert pianist during low-stress (rehearsal) and high-stress performance conditions (concert). The data confirmed that, during public performance, the musician experienced a heightened degree of physiological stress.

## Materials and Methods

### Aims of the Research

In the literature, several aspects of gesture and behavioral coordination among chamber musicians have been reported. However, there is a lack of research on combined music regulators and few studies examine how these communicative behaviors vary in different performance conditions. This research was developed considering the main functions of communicative behaviors in music ensemble performance, which have both a social – managing the interpersonal relationships within the group – and a musical dimension – contributing to create a whole and outstanding harmonic result from individual melodic lines. The purpose of this study was threefold; firstly to analyse how members of two string quartets employed music regulators in rehearsal (low stress performance condition, LSP) and concert (high stress performance condition, HSP) in ecological conditions; secondly to explore how eye contact, in terms of quantity and nature, varied alongside other music regulators as a function of LSP and HSP; and finally to consider what influence the nature of the musical piece had on the interactions between performers in these two conditions.

### Participants

Two string quartets composed of advanced conservatoire-level performers, who are active on the international chamber music scene, participated in the research project. The ensembles were *Quartet A* and *Quartet B* (names have been changed to preserve anonymity). All members of the two ensembles completed their conservatoire training in France and the UK. *Quartet A*, comprising two male and two female (second violin and cello) members, is based in France, and its members had played together for 7 years at the time of the study. *Quartet B*, with three male and one female (first violin) members, is based in the UK and was founded in 2002: the musicians had played together for 9 years at the time of the study. Both ensembles have won several important international prizes, and their professional activity includes musical performances all around Europe (UK, France, Austria, Italy, and so on). This study was carried out in accordance with the ethical guidelines of the British Psychological Society with written informed consent from all participants in accordance with the Declaration of Helsinki.

### Procedure

Video recordings of two performances were made: a low-stress performance (LSP), recorded in a rehearsal setting, and a high-stress performance (HSP), recorded in a live concert setting. Members of the two ensembles were informed that the video recordings would be used for research purposes only. They were given a general explanation of the purpose of the investigation with no specific research hypothesis or detailed information provided at the outset. Participants were given the opportunity to view the videos after the performances. In order to confirm that the LSP was in fact low stress and that the concert was high stress, participants were asked to complete the 20-item Spielberger State Anxiety Inventory before both conditions. These data confirmed the expected direction and magnitude of state anxiety experienced (i.e., that it was higher in the HSP; see beginning of the results section).

For both ensembles, one polished run-through of a set program in a typical rehearsal space and the concert performance of the same program were video recorded. The video recordings were shot in ecological conditions: that is, the rehearsals and concerts were authentic situations (Biasutti, 2012, 2015a,b) rather than artificial experimental tasks. The context was a European master class on string chamber music, which was attended by both ensembles. While this condition provided ecological validity to the research, it also had some limitations: the differences in the repertories played by the two groups were due to the program requirements of the master class.

The rehearsal performances took place in an appropriate room in the music school in Fiesole (Italy), while the concerts were organized as master final exhibitions. The final performances took place in the Chiostro of San Salvatore al Monte (Florence, Italy) in front of an audience of about 60 people.

*Quartet A* performed Béla Bartók’s Op.17 n°2 (Movements I. Moderato; II. Allegro molto capriccioso; and III. Lento). *Quartet B* performed Franz Joseph Haydn’s Op.77 n°1 (Movements: I. Allegro moderato; II. Minuetto, Presto; III. Andante; and IV. Vivace Assai). Due to the different total durations of the pieces performed, it was decided to analyse similar durations of video recording per group according to selected gestures. For *Quartet A*, video recordings of the first movement (Moderato) performances were considered, while for the *Quartet B*, video recordings of the first and the fourth movements performances were examined. This choice was made considering the duration of the movements: since an aim of the study was to maintain the ecological validity of the performance conditions, the researchers could not choose the repertoire to be played and, therefore, decided to examine ensembles’ performances within similar intervals of time (about ten minutes). Both ensembles reported similar levels of familiarity and preparation prior to the data collection sessions. **Table 1** presents detailed information about the pieces and durations of the movements performed.

**Table 1:** Time durations (in min, s.) of the analyzed performances.

Quartet	Author	Title	Year	Movements analyzed	Durations (min, s.) for LSP	Durations (min, s.) for HSP
*A*	Béla Bartók	Op.17 n°2 for two Violins, Viola, Cello	1915–1917	1. Moderato	10′ 06″	11′ 00″
*B*	Franz Joseph Haydn	Op.77 n°1 for two Violins, Viola and Cello	1791	1. Allegro Moderato 4. Finale Presto Total	5′ 51″ 3′ 48″ 9′ 39″	6′ 28″ 3′ 49″ 10′ 17″

*HSP, high stress performance; LSP, low stress performance.*

### Apparatus

The following two video cameras were used for video recording the sessions: sony DSR-PD150P and Canon DM-XM1E. The two video cameras were mounted on tripods and captured a fixed shot of all members of the ensemble from different angles. One video camera was posed specifically for capturing the faces of viola and cello performers, while the other for capturing specifically the faces of first and second violin performers. In this way it was possible to ascertain during the video analysis whether eye contact had actually been made or not between two individuals. A technician was in charge to make the video recordings and was able to check on these cameras – to make sure that any inconvenience would not be incurred (e.g., video camera gone out of focus for some reason, or other problems). At the end of the video recordings the data were saved to a computer and mounted for video analysis. Video cameras were placed in the same angular orientation to the center stage during the rehearsal, and the concert and the process of video capture was replicated across the two conditions.

## Results

In the results section, the data of LSP and HSP comparison for anxiety are reported at the beginning to support the investigation of performance across the two conditions. After, a description of the session is reported for accounting the processes that occurred during string quartets’ performances. The following analyses regard the number of attacks (including attack emphasis) and eye contact during LSP and HSP performances. In addition, other qualitative aspects, such as the kind of eye contact, who performed the eye contact and its direction, are considered. Statistical analyses were performed using IBM-SPSS (Version 22).

### LSP and HSP Comparison for Anxiety

All four members of each quartet ensemble were tested with the 20-item Spielberger State Anxiety Inventory (Spielberger et al., 1983), and their scores were calculated. Anxiety scores before the LSP and the HSP were as follow: LSP mean score = 36.5 (*SD* = 4.44), HSP mean score = 49.72 (*SD* = 9.63). To better understand anxiety scores previously reported, scores of 36.47 (*SD* = 10.02) indicate moderate levels of anxiety for young men and 38.76 (*SD* = 11.95) for young women (Spielberger et al., 1983). The data were analyzed using a Wilcoxon Test. A non-parametric test was chosen because the sample includes a small number of participants. The results show a significant difference of anxiety scores in the two conditions with *z* = –2.383, *p* = 0.05. There was a significant increase in state anxiety in the HSP, indicating that the concert performance condition used in this study was indeed high stress. Before embarking on a detailed categorisation of bodily gestures for articulating attacks and eye contact according to the LSP and HSP conditions, it is instructive to look first at a preliminary analysis of the rehearsals and concerts, in terms of processes that occur during string quartet performance that cannot be accounted for in quantitative terms.

### Description of Rehearsals and Concerts

This description includes the researchers’ perceptions about musical, social and communicative dynamics that developed in the performance contexts. It is based on the work carried out on the video recordings in which eye contact and gestures for articulating attacks were considered. The roles of the musicians in the two groups were well established. They were aware of the moments of greatest difficulty and expressiveness of the pieces and were able to control the performance speed from LSP to HSP in relationship to the aimed expressive dimension.

In the case of *Quartet A*, the concert lasted longer than the rehearsal, perhaps for the need to find a more expressive dimension due to the presence of the audience. There was an increase of the communicative strength of the cello compared with the other instruments during the concert. According to the musical score, she had to give several attacks, and the group often referred to her.

Also in the case of *Quartet B*, the speed of performance was slower for both the first (Allegro Moderato) and last movements (Finale Presto) during HSP than LSP. The first violin seemed conscious of her own movements, and she showed a great involvement in the overall performance especially during HSP: she seemed to move more decidedly than the others.

With regard to bodily gestures for articulations connected with attacks, they were influenced directly by the high stress condition and presence of the audience, conveying both communicative and expressive meanings. The gestures which characterized the attack seemed to have a double function, connected both with expressive and communicative dimensions of music performance in the two groups. Attacks emphasize sound production, conveying an expressive sense of start of the musical speech.

With regard to eye contact, different kinds were detected during LSP and HSP. Two main functions emerge for eye contact: communication between ensemble members for emotional sharing and monitoring group performance. Eye contact appears related to the score, as it is used to support synchronization, especially in critical technical or rhythmical passages.

### Detailed Analyses

The main aim of the reported analyses is to determine the number of gestures for articulating attacks (including their emphasis) and eye contact during LSP and HSP performances. In addition, other qualitative aspects such as the kind of eye contact, who performed the eye contact and its direction are considered.

#### Analysis of Attacks and Attack Emphasis

To determine the performance attacks, the following definition was used: the attack is an intentional gesture enacted by one performer addressed to other members with the aim of synchronizing the performance. This definition is consistent with that provided by Randel (2003) in the *Harvard Dictionary of Music*:

“*Attack: the characteristics of the beginning of a sound either as described technically by the science of acoustics or more loosely as a function of articulation in performance; also the degree of precision with which members of an ensemble coordinate the beginning of pitches.*” (Randel, 2003, p. 62).

Bodily gestures involved in attacks are connected with the articulations of the musical piece and with the frequency with which ensemble musicians need to coordinate their performances. In the current study, the researchers have chosen to focus on emphasis of attack gestures, as observable behaviors.

In order to verify the consistency of the attacks, a score was computed to assist confirmation of an attack versus an expressive performance gesture (i.e., movements of the arm with the bow to emphasize a musical accent or a group of beats). Two observers analyzed independently the video recordings to identify the total number of attacks using this definition. Agreement about their coding was about 88% of 34 observations. The two observers identified discrepancies in their coding and, through a criteria based discussion, agreement was reached on classification. In sum, there were 17 attacks for *Quartet A* and 17 for *Quartet B* (nine attacks for the first movement and eight for the fourth movement). For both ensembles, the attacks were made reliably at the same locations in the music for the LSP and HSP conditions.

From the video recordings, sequences of the identified attacks were selected, cut and edited randomly in four new videos per ensemble. For each ensemble, the first video was used as a trial and included 5 randomly chosen attack sequences, while the other 3 videos included all the 34 video sequences of attacks (17 attacks from the LSP condition and 17 from the HSP condition) in random order. Nine independent observers were asked to evaluate the emphasis of each attack, watching the eight videos and using a 5-point Likert-type scale (1 = minimum level of emphasis: limited movements of the head and the arms, scarce or minimum control by means of eye contact, minimum accents at the beginning of the musical phrase; 5 = maximum level of emphasis: broad movements of the head and the arms, involvement of facial expressions, eye contact oriented to colleagues, relevant accents at the beginning of the musical phrase). The observers were all expert music performers, mainly on string instruments.

The first step of data analysis was to evaluate the level of agreement among the nine observers which was assessed with Kendall’s W coefficient. Since *W* = 0.65 (*p* < 0.01), it can be assumed that there is a significant level of agreement among raters. The second step was to calculate a mean score for each attack and to calculate a paired-samples *t*-test, pooling the data from the two ensembles. *T*-test results showed that there was a significant difference between attack emphasis in the LSP condition and the HSP condition (*t*_(33)_ = 4.29, *p <* 0.01; for LSP condition: *M* = 2.38, *SD* = 0.62; for HSP condition: *M* = 2.78, *SD* = 0.78).

#### Analysis of Eye Contact

To determine the total amount of eye contact, the following definition was used: eye contact is intentional eye movement towards one or more performers with the aim of checking some aspects of the performance, such as synchronizing bow movement, intensity, and gesture. Instances of eye contact were counted for each performer.

Two observers worked from identical video footage in the first stage. The observers had to identify points of eye contact, discussing their consistency and checking for ambiguous cases. For *Quartet A* the final eye contact agreed values were 69 for LSP and 58 for HSP: these values include only points of eye contact which were confirmed and accepted by both observers. Several instances of eye contact did not match between the LSP and HSP conditions and were performed at different locations in the music. The same process was applied to data observed from the *Quartet B*. In sum, 67 instances of eye contact were identified for LSP and 70 for HSP for the first movement, and 30 for LSP and 41 for HSP for the fourth movement of the *Quartet B* (total 97 for LSP and 111 for HSP).

**FIGURE 1:**
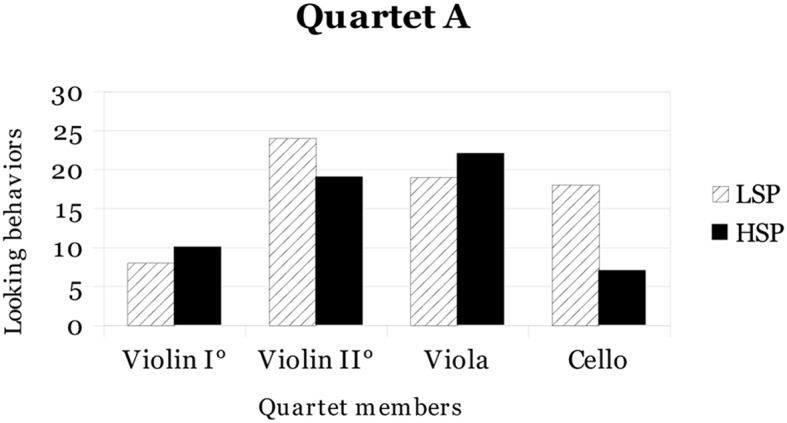
**Total number of instances of eye contact in low stress performance (LSP) and high stress performance (HSP) for each member of *Quartet A***.

**FIGURE 2:**
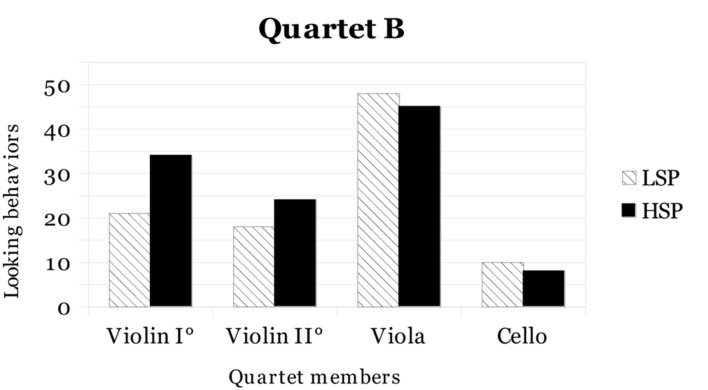
**Total number of instances of eye contact in LSP and HSP for each member of *Quartet B***.

Another analysis was conducted in order to verify whether the eye contact was addressed by the same or different performers during LSP and HSP conditions. *Quartet A* and *Quartet B* results for eye contact in the two conditions (LSP and HSP) are shown in **Figures 1** and **2**, respectively.

As shown in the figures, *Quartet A* first violin and viola and *Quartet B* first and second violins increased eye contact from LSP to HSP, while the other performers decreased eye contact. In both ensembles, it seems that members who assumed a leading role (e.g., the first violinists) showed an increase in eye contact from LSP to HSP (respectively, 8 versus 10 for *Quartet A* first violin, and 21 versus 34 for *Quartet B* first violin). However, the increase of *Quartet B* first violin was markedly more evident than the *Quartet A* first violin, probably for her more prominent role as described in “Description of rehearsals and concerts” above. Data about eye contact were examined performing both quantitative and qualitative analyses.

For the quantitative analyses, the overall frequencies of eye contact divided across ensemble roles (Violin I°, Violin II°, Viola, and Cello) in the two performance conditions (LSP and HSP) were considered and a Chi-square test was performed: according to the results, no significant differences were found.

Considering the qualitative analyses, different types were detected including the moments when they were enacted and the responses of the other performers in terms of glancing back. One could surmise that the performers used glances directed to colleagues with different meanings and purposes. Many instances share similar characteristics in both ensembles, and some visual exchanges were more frequent. A follow-up categorisation was conducted in order to identify specific communicative patterns including the directions of glances; the behavior of all the performers before the considered eye contact; the events that follow eye contact, in terms of behavior of the performer who took the glance and the colleagues’ responses. The eye contact detected during LSP and HSP between the two ensembles was of the following three main types:

*one-direction eye contact*, when one player looked to only one colleague for purposes such as performance synchronization, monitoring expressive or visual aspects of performance, without receiving any response by the observed person.

*“glanced back” eye contact*, when one player looked at one colleague who in turn replied with another glance. These exchanges were usually connected with seeking for feedback on individual performance or looking for emotional sharing;

*multiple-direction eye contact*, when one player, without glancing down or looking away, looked directly from one colleague to another. These visual signalings had often a monitoring function because they were more frequent when a member had to start a musical phrase and wanted to be sure that other performers were ready for that.

An additional quantitative analysis was performed considering the number and the direction of eye contact. Some differences emerged between the LSP and HSP conditions as can be seen in **Tables 2** and **3**. There are some recurrent patterns: for both ensembles, most of the second violinist’s glances during rehearsal was directed towards the violist, while the cellist, in general, looked mainly to the first violinist. First violinists showed a more diversified pattern, a behavior which may be influenced by several factors, probably both personal and professional.

**Table 2:** Direction and frequency of eye contact for each member of *Quartet A*.

Direction	Violin I		Direction	Violin II	
	LSP	HSP		LSP	HSP
Violin II	1	0	Violin I	0	6
Viola	3	2	Viola	18	4
Cello	3	8	Cello	6	7
Cello–Viola–Violin II	1	0		

**Direction**	**Viola**		**Direction**	**Cello**	
	**LSP**	**HSP**		**LSP**	**HSP**

Violin I	11	7	Violin I	8	5
Violin II	6	12	Violin II	9	2
Cello	2	3	Viola	0	0
			Violin II–Violin I	1	0

*HSP, high stress performance; LSP, low stress performance*.

**Table 3:** Direction and frequency of eye contact for each member of *Quartet B*.

Direction	Violin I				Direction	Violin II			
	Allegro Moderato		Finale Presto			Allegro Moderato		Finale Presto	
	LSP	HSP	LSP	HSP		LSP	HSP	LSP	HSP
Violin II	0	2	0	2	Violin I	4	2	2	2
Viola	6	8	3	6	Viola	10	12	2	2
Cello	7	10	3	4	Cello	0	3	0	3
Violin II–Viola	1	0	0	0					
Viola–Violin II	0	1	0	0					
Cello–Viola	1	0	0	1					

**Direction**	**Viola**				**Direction**	**Cello**			
	**Allegro Moderato**		**Finale Presto**			**Allegro Moderato**		**Finale Presto**	
	**LSP**	**HSP**	**LSP**	**HSP**		**LSP**	**HSP**	**LSP**	**HSP**

Violin I	7	3	13	5	Violin I	6	3	3	3
Violin II	13	16	4	11	Violin II	1	1	0	0
Cello	11	6	0	2	Viola	0	1	0	0
Violin I–Violin II	0	1	0	0					
V II–V I–V II	0	1	0	0					

*HSP, high stress performance; LSP, low stress performance*.

As can be seen in the tables, there was large variability in eye contact between LSP and HSP both in number and direction. These data suggest that eye contact was not standardized during the two performances, but changed according to the context.

## Discussion

This study has focused on two kinds of music regulators: bodily gestures for articulating attacks and eye contact. The aim was to understand how these regulators are used by quartet members and whether differences emerge between a low stress performance (LSP), recorded during a rehearsal, and a high stress performance (HSP), recorded during a concert, considering the impact that stress and music performance anxiety may have in ensemble communication. Two different string quartet ensembles were used in the research: each ensemble was composed of different expert musicians. Each group played a specific composition from two different eras. The study was conducted under ecologically valid conditions as part of a European master class program in string chamber music. Videos of the two string quartet performances were analyzed by two observers and the evaluation of attack emphasis was made by nine expert evaluators. There was a significant increase in state anxiety in the HSP, indicating that the concert performance achieved the objective of providing a higher stress situation than the rehearsal, confirming what Williamon et al. (2014) and Fancourt et al. (2015) have found on high-stress and low-stress performance conditions. This outcome supports the research findings by LeBlanc et al. (1997) who found a significant increase of self-report perceived anxiety in a performance condition with an audience.

With regard to the temporal nature of attacks, while they demonstrated a standardized pattern, occurring in the same locations during both LSP and HSP, the findings provide evidence of a significant difference in the emphasis between the LSP and HSP conditions. This is in agreement with results by Davidson and Correia (2001) who found a higher emphasis of performer’s gestures during real performance conditions. In these situations, there is also a role of stress and perceived anxiety, which may influence the physiological and physical dimension of music making, causing variations in the way musicians usually face performance (Lehmann et al., 2007). Thus a dual role for gestures for articulating attacks may be posited: as expressive and communicative movements (Davidson and Correia, 2002; Davidson and King, 2004). The importance of attacks both in the auditory and the visual dimensions was underlined: they affect sound production, but they may also be considered as a visual bond that links co-performers’ activity and connects the musicians with their audience.

With regard to the frequencies of instances of eye contact, the Chi-square test showed no significant differences in the two performance conditions (LSP and HSP). A qualitative analysis indicated variability in the number and the direction of eye contact in the two quartets (for *Quartet A*, 69 LSP versus 58 HSP, and for *Quartet B*, 97 LSP versus 111 HSP). Eye contact may be also influenced by the kind of music and the different challenges presented by each piece. The degree of difficulty and the addition of HSP combine to increase the performers’ attention to the technical aspects of music narrowing their focus and restricting eye contact. This may be explained by the Haydn group (*Quartet B*) reporting fewer technical difficulties in the piece and thus being able to play more expressively with interactions between co-performers (Davidson and Correia, 2001). The evidence from the attack emphasis data supports this distinction in task demands. For both ensembles the first violin increased the amount of eye contact with other quartet members during HSP than LSP. However, this is less evident for *Quartet A* (8 versus 10 instances) than *Quartet B* (21 versus 34 instances) probably linked to the greater extroverted role for the violin in the work by Haydn. The marked shift in eye contact supports both the freeing of technical attention and the communication of ensemble togetherness that might enhance audience connection. This behavioral pattern is consistent with the typical leading role in string quartet ensembles adopted by the first violin identified by King (2006). The role of leader is to help synchronise their co-performers, placing upon them a particular responsibility during quartet performance which becomes more relevant in front of an audience. However, in contrast to King’s (2006) view, the cellist in the *Quartet A* demonstrated a ‘co-leading’ role, supporting the first violin in the synchronization of the task. The reason for this may be the characteristics of the piece being performed because the Bartók piece emphasized the first violin part less through the use of dialog between the instruments rather than a melodic line with accompaniment. The Bartók data showed that no instrument prevailed over the others but rather a transfer of the theme between the instruments without big movements or gestures. Probably, gestures may be influenced by the kind of music, and the Bartók is known to be a difficult piece. This finding would confirm Davidson and Correia’s (2002) observation of a difference between two pieces of different eras. However, one reason for our observation may be the novel use of two separate ensembles, though further work could compare various ensembles across different kinds of repertoire in rehearsal and concert to demonstrate the consistency of this behavior.

In observing the *Quartet B*, the amplitude and energy of first violinist’s gestures were recognized: probably her gestures had a significant influence on the others, who followed her and were conducted by her. These findings are consistent with Davidson and Good’s (2002) research on the social and musical dimensions of coordination in string quartet ensemble performance. Further research is needed to investigate these reflections in greater depth in order to verify how the leading role could be influenced by musical and personal/group characteristics.

With regard to eye contact patterns, visual exchanges between the first violin and the cellist and between the second violin and the violist seem the most important and are similar to those found by Davidson and Good (2002). This supports the idea that eye contact has an important role in ensemble communication. Findings from the analysis of qualitative aspects of eye contact reflect the categorisation made by Kurosawa and Davidson (2005) on the functions of behaviors during performance. One-direction eye contact seems to have a self-regulatory function; multiple-direction eye contact shows mainly a function of connection with co-performers, while “glanced-back” eye contact represents a function of communication inside the group and sharing of musical and personal meanings.

## Conclusion

This study shows the fundamental role played by non-verbal communication within string quartets in an ecological context, whether performing in low or high stress conditions. The findings provide evidence of behavioral coordination among chamber musicians and show that, during musical performance, gestures for articulating attacks seem to be influenced more by the presence of the audience, conveying both communicative and expressive meanings. Conversely, eye contact has two important functions for coordinating the ensemble: communication between quartet members, and monitoring group performance. Eye contact appears related to the score, as it is used to support synchronization, especially in critical technical or rhythmical passages, hence the differences observed between Bartók (A) and Haydn (B).

It seems that attacks are standardized since they occurred in the same locations during LSP and HSP. Attack emphasis is mediated by the situation, because there was a significant difference in expressive attack gestures during LSP and HSP. In addition, there was variability for eye contact from LSP to HSP considering characteristics such as who performed the eye contact and the direction of the eye contact. These data provide evidence that eye contact is not standardized and is conditioned by idiosyncratic performing situations. The approach of the current research has restrictions because of the limited number of participants and the limited segments of music considered. More specifically, the ecological condition represented by the attendance of the two ensembles in a European master class in string chamber music meant that it was not possible to be prescriptive in the pieces used, which limited comparison between ensembles. While this situation improved the ecological validity to the study it did limit the control over the performances and over possible confounding variables. Issues connected with the internal validity of this study, due to the limited control over the study conditions, have to be taking into account while considering the current findings. In addition, the analysis focuses on selected aspects that can be related to the level of stress experienced during the performance; there are other factors that have not been directly addressed here (e.g., variations in speed during rehearsal and during performance) but that could be considered in a future development of the research. However, the results are a platform for developing a future research plan involving a larger number of ensembles, and employing quantitative data collection techniques and related statistical analyses. Future research may also consider the role of movement and gesture in different musical styles, such as jazz and classical music, discovering possible similarities and differences.

### Educational Implications

The results may be used to improve an understanding of body movement and gesture while performing in chamber ensembles. From these findings, further research development can be planned, considering more in detail the development of gesture in relation with the increase of the stress level during the whole learning process. Since in music education, interpersonal and communicative abilities implied in ensemble performance are not directly taught to students (Lehmann et al., 2007), it seems important to understand the social processes on which this activity is based. Performing efficaciously in a string quartet is usually learned in a work environment which is characterized by implicit rules. To reflect on the implicit level of these processes and how eye contact and gestures for articulating attacks are used during rehearsals and performance is relevant for developing effective rehearsing and performing strategies. According to Davidson and King (2004), body gestures and eye contact are very important in ensemble performance, since they carry out a dual role of coordinating musical and social elements: they contribute to the uniformity and expressiveness of performance and also they help to manage the social context of the ensemble. To ensure these functions, gestures have to be clearly and rapidly decoded by co-performers in the chamber group; also eye contact needs to be unequivocally interpreted to transmit more effectively non-verbal information within the ensemble. Understanding how contextual conditions (e.g., performance anxiety) impact on these communicative elements may be useful to improve non-verbal communication between co-performers. Specific educational activities could be defined for these purposes since performers have to develop awareness and meta-cognitive strategies about the tasks involved during musical performance (Biasutti, 2013). A deeper analysis of non-verbal communication between co-performers may help teachers to address specifically these issues during ensemble coaching sessions in order to improve the quality of students’ learning and the overall performance.

### Implications for Further Research

The current study was developed with an emphasis on ecologically validity and applied an experimental design to analyse the gestures for articulating attacks and the eye contact in two different performance stress conditions. The results support the need for further research in non-verbal communication in ensemble performance. The study contributed in setting criteria for scoring attack and eye contact, checking inter-rater reliability and validating the procedure. The method used in the current research could be extended to investigate differences between musical expressive gestures in high- and low-stress situations, and to develop existing research on the self- versus other-regulatory functions of eye contact and bodily gestures in quartet rehearsal and performance. Future studies should focus in detail on these two kinds of regulators, examining the impact that emotional expression, time synchronization, and accuracy for articulations have on their display. One possibility is the extension of the research to other performance gestures and to other musical expressive behaviors. It would be instructive to investigate how such behaviors impact performance, and whether there are differences between rehearsal and concert contexts. In the current research, emphasis of gestures for articulating attacks were different in LSP than HSP conditions, and similar findings may be found for other expressive gestures. Further research could adopt a prescriptive approach to the music performed, using one or more genres across two or more quartets to examine any variation in communicative behaviors while under low and high stress scenarios. Another possibility is to develop the research to triangulate qualitative observations with other data sources, particularly those of physiological data collection devices, such as the Zephyr BioHarness which come equipped with in-built accelerometers. Such investigation would allow behavioral data to be matched against corresponding physiological responses to LSP and HSP, especially HRV, which are known to change dramatically under conditions of high performance stress (Williamon et al., 2013).

## Author Contributions

MB was the idea originator of the paper, contributed with the data analysis method, results interpretation and wrote the paper; EC contributed with the literature review and analysed the data ; DW decided the method of study, collected the data and revised critically the paper; AW was the director of the research project, decided the method of the study, collected the data, coordinated the activities and revised critically the paper.

## Conflict of Interest Statement

The authors declare that the research was conducted in the absence of any commercial or financial relationships that could be construed as a potential conflict of interest.
